# The crystal structure of human transport and Golgi organization 2 homolog (TANGO2) suggests a cysteine N-terminal nucleophile (Ntn) hydrolase

**DOI:** 10.1107/S2059798326001968

**Published:** 2026-04-01

**Authors:** Dayong Zhou, Lirong Chen, John Rose, Bi-Cheng Wang

**Affiliations:** a Department of Biochemistry and Molecular Biology University of Georgia Athens GA30602 USA; McGill University, Canada

**Keywords:** TANGO2, TDD, Ntn-hydrolases, heme, pathogenic variants, X-ray crystallography

## Abstract

Recently, there has been growing interest in the function and physiological importance of human TANGO2 (transport and Golgi organization 2 homolog), particularly whether it acts as a heme-trafficking protein. To address this question, we experimentally determined the three-dimensional structure of TANGO2. Our crystallographic analysis indicates that interactions between heme and TANGO2 are nonspecific. Structural comparison of the TANGO2 crystal structure with known cysteine Ntn-hydrolases allowed us to identify a putative active site, catalytic residues and a substrate-binding cavity that correspond to residues that are mutated in pathogenic TANGO2 variants. Based on these features, we propose that TANGO2 may utilize fatty-acid derivatives as substrates, suggesting a potential role in lipid metabolism. Mutations in the human TANGO2 gene cause TANGO2 deficiency disorder, a multisystem, life-threatening disease with onset in early childhood. Together, our results provide new insights into the molecular function of TANGO2 and help to resolve the ongoing debate regarding whether it functions as a heme-trafficking protein.

## Abstract 2

This study reports the first crystal structure of human TANGO2, mutations of which are responsible for TANGO2 deficiency disorder. The crystallographic analysis demonstrates that interactions between heme and TANGO2 are nonspecific, helping to address ongoing questions about the role of TANGO2 as a heme-trafficking protein.

## 1. Introduction

TANGO2 deficiency disorder (TDD) is a rare autosomal recessive disease caused by mutations in the human TANGO2 gene. It is characterized by complex, often life-threatening, clinical symptoms that appear early in childhood, including episodic rhabdomyolysis, global developmental delay, seizures, encephalopathy, cardiac arrhythmias and acute metabolic crises (Miyake *et al.*, 2023 ▸; Kremer *et al.*, 2016 ▸;Jennions *et al.*, 2019 ▸; Milev *et al.*, 2021 ▸; Powers, 2024 ▸). Some studies suggest that TANGO2 may play a role in lipid metabolism and heme trafficking. However, the function and physiological significance of TANGO2 remain unclear.

TANGO2, first identified in 2006, is crucial for the transport and organization of the Golgi complex in *Drosophila* (Bard *et al.*, 2006 ▸). A recent study using time-lapse confocal microscopy in human HepG2 and U2OS cells showed that mScarlet-tagged TANGO2 is located within the mitochondrial lumen (Lujan *et al.*, 2025 ▸). Similar results have reported that red fluorescent protein (RFP)-tagged TANGO2 was found in the mitochondrial-enriched fraction. Additionally, the knockout or mutation of TANGO2 in human cells can alter the mitochondrial morphology (Milev *et al.*, 2021 ▸; Stentenbach *et al.*, 2025 ▸). Mitochondrial energy metabolism disorders characterize the clinical and biochemical phenotypes of TDD patients. Proteomic signature analysis of fibroblasts from TDD patients revealed significant changes in proteins involved in the ER–Golgi network, mitochondria, amino-acid metabolism and, in particular, fatty-acid oxidation (Mingirulli *et al.*, 2020 ▸; Asadi *et al.*, 2023 ▸). Lipidomics analysis revealed that TANGO2 mutations result in a significant increase in lysophosphatidic acid (LPA) and a concomitant decrease in its biosynthetic precursor phosphatidic acid (PA), suggesting that TANGO2 is likely to be involved in acyl-CoA metabolism. As a result, TANGO2 can affect the metabolism of various fatty acids and alter the overall lipid composition, leading to increased levels of reactive oxygen species and lipid peroxidation (Lujan *et al.*, 2023 ▸; Mehranfar *et al.*, 2024 ▸). Recent studies have shown that TANGO2 is an acyl-CoA binding protein capable of directly interacting with the lipid portion of acyl-CoA molecules in the TANGO2 putative binding pocket. Thus, TANGO2 may function as a crucial shuttle protein for the intracellular trafficking of acyl-CoA in lipid metabolism (Sandkuhler & Mackenzie, 2025 ▸; Lujan *et al.*, 2025 ▸). Meanwhile, another recent study (Heiman *et al.*, 2022 ▸) specifically linked TANGO2 deficiency to mitochondrial dysfunction, showing that mitochondrial bioenergetics are strongly affected by TANGO2 mutations, impairing fatty-acid oxidation and oxidative phosphorylation, as shown by abnormal mitochondrial respiration rates, reduced ATP production and decreased expression of mitochondrial proteins.

Besides the speculation that TANGO2 may play a crucial role in lipid metabolism and mitochondrial ATP production, two recent studies suggest that human TANGO2 functions as a heme chaperone, which is essential for heme trafficking in eukaryotic cells (Sun *et al.*, 2022 ▸; Han *et al.*, 2023 ▸). Human TANGO2 can bind both ferrous and ferric heme, with dissociation constants (*K*_d_) of 15.4 and 38.7 µ*M*, respectively. Bacterial homologs show similar affinities *in vitro*. Moreover, TANGO2 is capable of heme transfer alone, and no other mitochondrial membrane proteins are required. However, to date, the function and physiological role of TANGO2 still remain undetermined, and more information is needed to fully understand its functions and the mechanism behind the related disorder. In early 2023, we determined the first crystal structure of human TANGO2 at a resolution of 1.53 Å using X-ray crystallography, and we deposited its coordinates in the Protein Data Bank (PDB) in May of that year. They have been available to the community since June 2024 (PDB entry 8sv7). The crystal structure of TANGO2 reveals a previously predicted four-layered αββα fold, which was also recently confirmed by Cooper *et al.* (2025 ▸).

In this study, we reveal significant similarities between TANGO2 and cysteine N-terminal nucleophile hydrolases (Ntn-hydrolases). By comparison between the structures of TANGO2 and known cysteine Ntn-hydrolases, we identified the potential active site, catalytic residues and substrate-binding cavity of TANGO2, which correspond to the mutated residues found in the pathogenic TANGO2 variants. The possible role of TANGO2 as a cysteine Ntn-hydrolase could aid in understanding the pathogenic mechanism of the disease. Additionally, our results indicate that TANGO2 may utilize fatty-acid derivatives as potential substrates by analyzing its substrate-binding cavity and structural models of TANGO2 complexed with fatty-acid substrate mimics, highlighting its possible roles in lipid metabolism. Finally, we experimentally explore whether TANGO2 functions as a heme-trafficking protein.

## 2. Experimental methods

### 2.1. Protein expression, production and purification

*Escherichia coli* strain BL21(DE3) pLysE was transformed with an expression plasmid (pET-28a-TANGO2-His) containing a C-terminal 6×His-tag and TEV protease cleavage site. A fresh colony was inoculated into 50 ml LB medium with 50 µg ml^−1^ kanamycin and grown aerobically at 37°C overnight. The entire overnight culture was then used to inoculate 1 l LB medium supplemented with 50 µg ml^−1^ kanamycin and grown at 37°C with shaking (250 rev min^−1^). When the OD_600_ reached 0.5, recombinant human TANGO2 expression was induced with 0.1 m*M* isopropyl β-d-1-thio­galactopyranoside (IPTG) and cultivation continued at 18°C for 16 h. The cells were harvested by centrifugation at 6000*g* for 15 min at 4°C and the cell pellet was resuspended in 30 ml lysis buffer consisting of 20 m*M* phosphate buffer pH 7.4, 500 m*M* NaCl, 20 m*M* imidazole, 10 µg ml^−1^ phenylmethylsulfonyl fluoride (PMSF). The cell suspension was sonicated on ice and clarified by centrifugation at 25 000*g* for 1 h. The supernatant was loaded onto an ÄKTApure system (Cytiva) with a 5 ml HisTrap HP column pre-equilibrated with binding buffer (20 m*M* phosphate buffer pH 7.4, 500 m*M* NaCl, 20 m*M* imidazole). His-tagged TANGO2 was eluted using a linear imidazole concentration gradient from 20 to 250 m*M*. The fractions containing TANGO2 were pooled and His-tagged TEV protease [1:50(*w*:*w*)] was added, followed by dialysis overnight at 4°C in 20 m*M* phosphate buffer pH 7.4, 150 m*M* NaCl. A second HisTrap HP column purification was performed to separate TANGO2 and cleaved 6×His tags. The tag-free TANGO2 was further purified by size-exclusion chromatography (HiLoad 16/60 Superdex 200, Cytiva) equilibrated with 20 m*M* HEPES pH 7.4, 1 m*M* DTT. Fractions containing purified TANGO2 were concentrated to approximately 15 mg ml^−1^ using 10 kDa molecular-weight cutoff Amicon concentrators (MilliporeSigma). The purity of the protein was analyzed by SDS–PAGE.

It is worth mentioning that we initially used an N-terminally His-tagged construct to express recombinant human TANGO2 in *E. coli*. However, purification with HisTrap column chromatography failed, even though SDS–PAGE showed TANGO2 overexpression in whole-cell lysates. As a result, we switched to the C-terminally His-tagged construct, which made protein expression and purification successful.

### 2.2. Crystallization and X-ray data collection of apo TANGO2

Crystals of apo TANGO2 were grown by the hanging-drop vapor-diffusion method at 291 K using 2 µl drops containing equal volumes of the protein solution and precipitant solution composed of 0.1 *M* bis-Tris pH 5.5, 1.0 *M* ammonium sulfate, 1% PEG 3350. Crystals appeared within two days and reached usable size in 4–5 days. The crystals were harvested and briefly immersed in a cryoprotectant solution consisting of precipitant with 15%(*v*/*v*) glycerol. The cryoprotected crystals were then flash-cooled and stored at cryogenic temperature for data collection. Due to the long unit-cell edges (*a* = 49.73, *b* = 49.96, *c* = 241.38 Å), initial diffraction data collected using home-source X-rays (200 µm beam size) showed severely overlapped diffraction spots. This issue was resolved by using small beam-size synchrotron X-rays, which allowed us to resolve the reflections for indexing.

The 1.53 Å resolution dataset was collected at 100 K on beamline 22-ID, SER-CAT, Advanced Photon Source (APS), Argonne National Laboratory using a 50 µm beam, an EIGER 16M detector and 0.979 Å wavelength X-rays. A total of 1440 images with 0.25° oscillations were collected at a crystal-to-detector distance of 230 mm and an exposure time of 0.2 s. The data were indexed, integrated and scaled using *HKL*-3000 (Minor *et al.*, 2006 ▸).

### 2.3. Structure determination and refinement

The structure of human TANGO2 was determined by molecular replacement (MR) using an *AlphaFold*2 (Jumper *et al.*, 2021 ▸) predicted model as the search model. In this study, the initial MR model from *phenix.phaser* was improved with *phenix.autobuild* (Liebschner *et al.*, 2019 ▸). Iterative cycles of refinement and manual model building were carried out using *phenix.refine* and *Coot* (Emsley *et al.*, 2010 ▸). The structural model was validated with the wwPDB Validation Server and deposited in the Protein Data Bank (PDB entry 8sv7). Data-collection and refinement statistics are summarized in Table 1 ▸.

### 2.4. Cavity calculations

The cavity calculations were carried out using the *KVFinder* package (Guerra *et al.*, 2021 ▸) in *UCSF ChimeraX* (Pettersen *et al.*, 2021 ▸). The coordinates of TANGO2 (PDB entry 8sv7), isopenicillin N *N*-acyltransferase (PDB entry 2x1d), bile-salt hydrolase (PDB entry 2hf0), human acid ceramidase (PDB entry 5u7z) and human *N*-acylethanolamine-hydrolyzing acid amidase (PDB entry 6dxx) were used for calculations, with default settings except that the minimum cavity volume was set to 50.0 Å^3^. Structural figures were prepared using *UCSF ChimeraX*.

### 2.5. Molecular docking

To understand the interactions between TANGO2 and putative substrate mimics, we used *AutoDock Vina* (Eberhardt *et al.*, 2021 ▸) in *UCSF Chimera* (Pettersen *et al.*, 2004 ▸) to dock three fatty acids to chain *A* of the crystal structure of TANGO2. The 3D structures of myristic acid (MYR), palmitic acid (PLM) and oleic acid (OLA) were downloaded from the PubChem database (https://pubchem.ncbi.nlm.nih.gov; CID 11005, 985 and 445639). The grid box measured approximately 45 × 45 × 50 Å and was set to cover the entire TANGO2 structure. The exhaustiveness of the search was set to 8, and the maximum energy difference was set to 3 kcal mol^−1^ for the docking calculation. Ligands such as 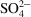 and water molecules in the crystal structure of TANGO2 were excluded. Ten binding modes were generated, and the mode with the lowest estimated binding energy (Δ*G*) was selected as the optimal docked model.

## 3. Results and discussion

### 3.1. The overall structure of apo TANGO2

Despite the recent publication of a human TANGO2 structure that closely resembles our findings, we consider it scientifically valuable to present our independently determined structure. Our results offer new insights that may refine current structural interpretations and contribute to a more comprehensive understanding of the function of TANGO2.

**Figure 1:**
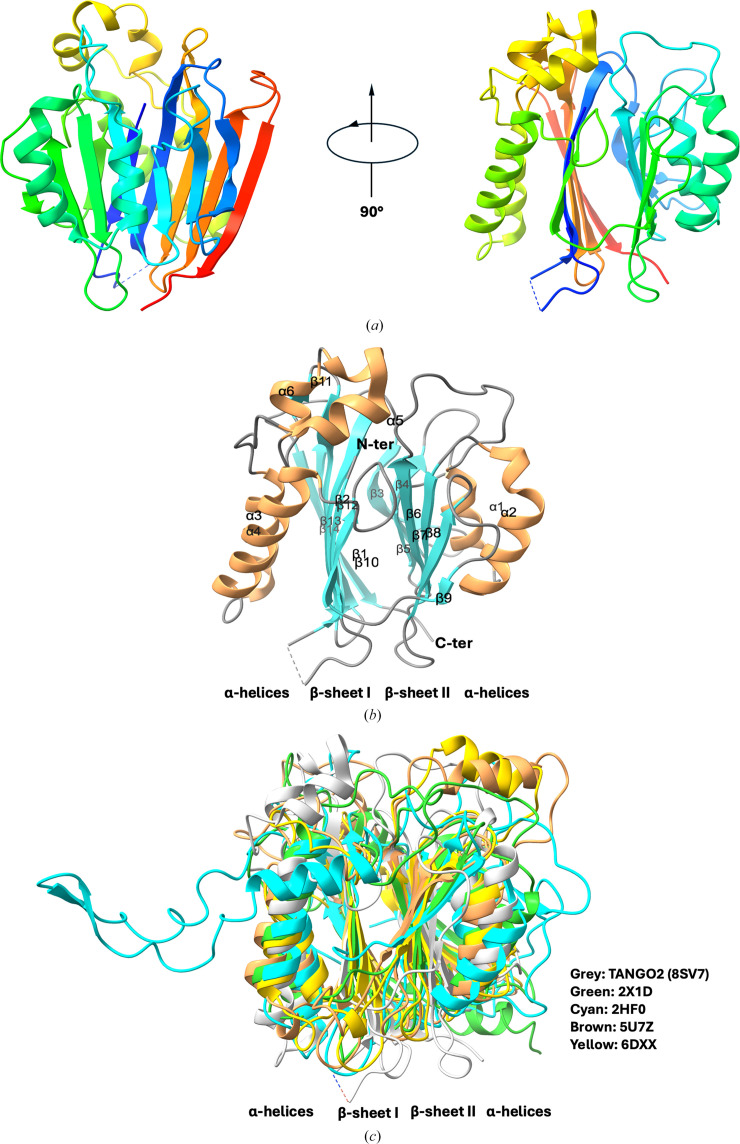
(*a*) Two orthogonal views of the TANGO2 structure. (*b*) Topology diagram of TANGO2. The annotated secondary-structure elements are colored using brown for helices (α1–α6), cyan for β-strands (β1–β14) and gray for coils. (*c*) The characteristic four-layered αββα fold of TANGO2 and structural superposition between TANGO2 (gray, PDB entry 8sv7) and four known cysteine Ntn-hydrolases, including penicillin acylase (green, PDB entry 2x1d), bile-salt hydrolase (cyan, PDB entry 2hf0), acid ceramidase (brown, PDB entry 5u7z) and *N*-acylethanolamine-hydrolyzing acid amidase (yellow, PDB entry 6dxx). The structural motifs (α-helices and β-sheets) are labeled to show the consensus αββα fold.

The overall TANGO2 structure is ellipsoidal, with approximate dimensions of 45 × 40 × 40 Å. The TANGO2 monomer folds into a single domain, comprising 14 β-strands, six α-helices and two 3_10_-helices (Fig. 1 ▸*a*). The crystal structure of TANGO2 adopts a characteristic four-layered αββα fold (two β-sheets sandwiched between two layers of α-helices; Carfi *et al.*, 1995 ▸). The TANGO2 αββα fold consists of 14 β-strands (β1–β14), forming two antiparallel sheets (sheets I and II) packed against each other and sandwiched by two α-helices: α1–α2 and α3–α6 (Fig. 1 ▸*b*). β-Sheet I contains the N- and C-termini and consists of seven strands (β1, β2 and β10–β14), while β-sheet II includes seven strands (β3–β9). Of these two sheets, sheet I is more flattened, and the two sheets are roughly parallel. Both β-sheets have predominant hydrophobic residues, with most of the hydrophobic side chains located at the interfaces between the β-sheets and the flanking α-helices.

**Figure 2:**
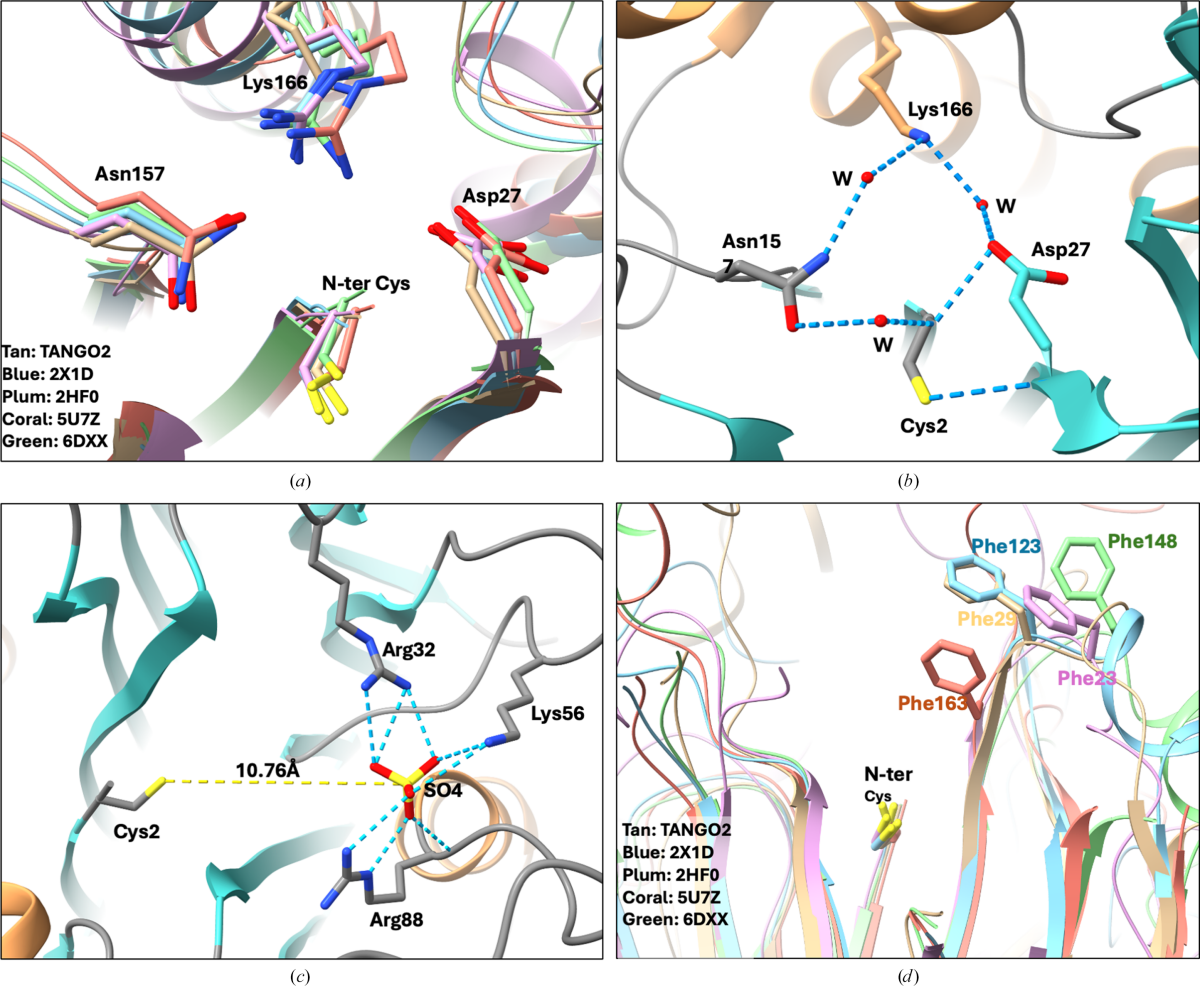
(*a*) Close-up view of the putative TANGO2 active site. The N-terminal Cys2 and three residues, Asp27, Asn157 and Lys166, are shown in stick representation. Superposition of conserved active-site residues in four known cysteine Ntn-hydrolases and TANGO2 with side chains colored tan (TANGO2, PDB entry 8sv7), blue (PDB entry 2x1d), plum (PDB entry 2hf0), coral (PDB entry 5u7z) and green (PDB entry 6dxx), respectively. Only TANGO2 residue numbers are shown for clarity. Lys166 in TANGO2 is replaced by arginine in all four cysteine Ntn-hydrolases. (*b*) The water-mediated hydrogen-bonding network of the putative TANGO2 active site. Hydrogen bonds are shown as blue dashed lines. (*c*) The sulfate ion (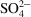) binding site of TANGO2. The anion is approximately 10.76 Å from the N-terminal Cys2 and coordinated by a triad of positively charged residues: Arg32, Lys56 and Arg88. (*d*) The conserved surface phenylalanine in TANGO2 and cysteine Ntn-hydrolases. Structural alignment shows that the surface-exposed Phe29 in TANGO2 is conserved across all four cysteine Ntn-hydrolases (Phe23 of PDB entry 2hf0, Phe123 of PDB entry 2x1d, Phe163 of PDB entry 5u7z and Phe148 of PDB entry 6dxx).

We have noticed that many studies utilize C-terminally tagged TANGO2 for *in vivo* cellular localization and *in vitro* biochemical assays (Milev *et al.*, 2021 ▸; Lujan *et al.*, 2023 ▸, 2025 ▸; Stentenbach *et al.*, 2025 ▸; Cooper *et al.*, 2025 ▸; Powers, 2024 ▸). The full-length TANGO2 consists of 276 amino acids; however, the N-terminal residue Met1 appeared disordered, as it was not visible in our 1.53 Å resolution electron-density map, whereas Cys2 was observed at the N-terminus. We hypothesize that the resulting N-terminal Cys2 may play a role in the structure and functions of the protein. Structural analysis of the Cys2 residue reveals that it is buried within the protein core and participates in a well defined hydrogen-bonding network. The α-amino group of Cys2 forms water-mediated hydrogen bonds with the side chains of Asp27 and Asn157, which are positioned on either side of Cys2. These interactions are likely to play a role in stabilizing Cys2 at its N-terminal location. Additionally, the side chain of Lys166 participates in a secondary hydrogen-bonding network with Asp27 and Asn157, also mediated by water molecules. Notably, the thiol side chain of Cys2 can form a hydrogen bond to the backbone of Asp27 (Figs. 2 ▸*a* and 2 ▸*b*).

Within the refined TANGO2 crystal structure, a sulfate ion (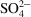) was identified approximately 10.76 Å from the N-terminal Cys2 (Fig. 2 ▸*c*). This anion is coordinated by a triad of positively charged residues: Arg32, Lys56 and Arg88. Given that the crystallization buffer contained 1 *M* ammonium sulfate, it is plausible that the sulfate ion originates from the crystallization conditions. Despite its likely nonphysiological origin, the presence of this sulfate ion raises intriguing questions about the functions of this binding triad. The spatial arrangement of positively charged residues suggests a potential role in stabilizing negatively charged ligands or intermediates. Further biochemical studies are necessary to determine whether this site contributes to TANGO2 functions.

### 3.2. Is TANGO2 a heme-transport protein?

Sun *et al.* (2022 ▸) demonstrated that TANGO2 functions as an intracellular heme-transport protein. However, recent studies (Sandkuhler *et al.*, 2023 ▸, 2025 ▸; Sacher *et al.*, 2024 ▸; Kim *et al.*, 2023 ▸; Jayaram *et al.*, 2025 ▸) still question whether TANGO2 truly acts as a heme-transport protein. After determining the apo TANGO2 structure, we soaked the crystals in heme solutions at various concentrations. To our delight, all soaked crystals exhibited light brown colors, indicating possible TANGO2–heme binding. Next, we performed co-crystallization with different heme concentrations. We analyzed 25 crystals by X-ray diffraction, including nine obtained by soaking and 16 by co-crystallization. Diffraction data for these light brown crystals were collected on beamline I04 at the Diamond Light Source in the UK during the 2023–2024 APS Upgrade shutdown. Although all of the crystals showed light brown colors, the electron-density maps did not reveal a specific heme location. These results suggest that the interactions between heme and TANGO2 are nonspecific and TANGO2 may not directly function as a heme-transport protein. Our crystallographic findings could help to resolve ongoing debates in this field.

### 3.3. Structural homolog search and alignment

Structural alignments using the *DALI* web server (Holm *et al.*, 2023 ▸) and *Foldseek* (van Kempen *et al.*, 2024 ▸) reveal that human TANGO2 shares structural similarities with four groups of known structures: (i) penicillin acylases (for example PDB entry 2x1d, 12% identity, *Z*-score 16.6; PDB entry 3pva, 10% identity, *Z*-score 16.5; Bokhove *et al.*, 2010 ▸; Suresh *et al.*, 1999 ▸), (ii) bile-salt hydrolases (for example PDB entry 2hf0, 9% identity, *Z*-score 15.7; PDB entry 7svh, 9% identity, *Z*-score 15.5; PDB entry 8blt, 10% identity, *Z*-score 16.5; Kumar *et al.*, 2006 ▸; Foley *et al.*, 2023 ▸; Karlov *et al.*, 2023 ▸). (iii) acid ceramidases (for example PDB entry 5u7z, 13% identity, *Z*-score 16.2; Gebai *et al.*, 2018 ▸) and (iv) *N*-acyl­ethanolamine-hydrolyzing acid amidases (NAAAs; for example PDB entry 6dxx, 12% identity, *Z*-score 16.4; PDB entry 6dxy, 12% identity, *Z*-score 16.8; Gorelik *et al.*, 2018 ▸) (Fig. 1 ▸*c* and Supplementary Fig. S1).

Despite their confusing names, all four groups of enzymes belong to the cysteine Ntn-hydrolase family, which is part of the Ntn-hydrolase superfamily. Identification of the Ntn-hydrolases largely depends on the characteristic αββα fold in their structures. All known Ntn-hydrolases are produced as inactive precursors that undergo activation by self-cleavage of the first N-terminal methionine or an internal peptide bond, exposing the catalytic N-terminal residue. This activation produces the mature enzymes, which catalyze nonprotein amide-bond hydrolysis via their newly exposed N-terminal cysteine, serine or, rarely, threonine (Gorelik *et al.*, 2018 ▸; Brannigan *et al.*, 1995 ▸). Although their sequence homology is low, all Ntn enzymes catalyze amide-bond hydrolysis. They show considerable variation in their substrate selectivity and specificity. Furthermore, these enzymes share similar catalytic residues and, therefore, catalyze substrate hydrolysis via a similar mechanism (Oinonen & Rouvinen, 2000 ▸; Linhorst & Lübke, 2022 ▸).

### 3.4. Structural comparisons with known cysteine N-terminal nucleophile hydrolases

With the high-resolution TANGO2 structure now available in this study, we compared the TANGO2 crystal structure with those of four known cysteine Ntn-hydrolases to investigate the potential role of TANGO2 as a cysteine Ntn-hydrolase and to identify its possible substrates.

#### 3.4.1. Comparison with *Penicillium chrysogenum*isopenicillin N *N*-acyltransferase (PDB entry 2x1d)

**Figure 3:**
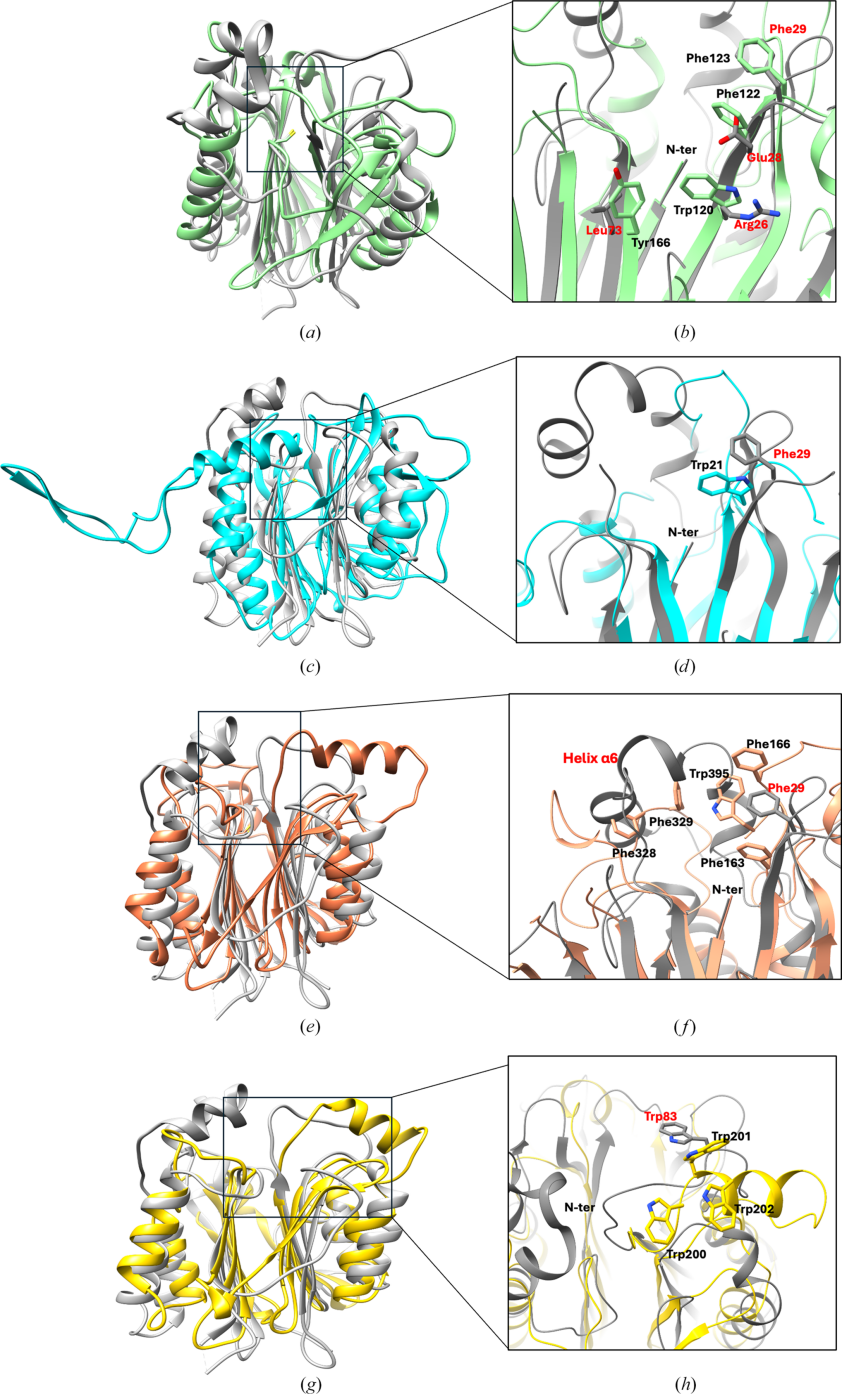
Structural comparisons between TANGO2 (gray, PDB entry 8sv7) and four cysteine Ntn-hydrolases. (*a*) Isopenicillin-N *N*-acyltransferase (green, PDB entry 2x1d). (*b*) A close-up view of the substrate-binding residues in AT (labeled in black) and their corresponding residues in TANGO2 (labeled in red). (*c*) Bile-salt hydrolase (cyan, PDB entry 2hf0). (*d*) Trp21 in BSH (labeled in black), important for substrate binding and selectivity, is replaced by Phe29 in TANGO2. (*e*) Acid ceramidase (brown, PDB entry 5u7z). (*f*) The hydrophobic substrate-binding residues of AC (labeled in black) are replaced by a short helix and Phe29 in TANGO2. (*g*) *N*-Acylethanolamine-hydrolyzing acid amidase (yellow, PDB entry 6dxx). (*h*) Three tryptophans in a ‘WWW’ segment (labeled in black), important for substrate binding, are replaced by Trp83 in TANGO2.

The isopenicillin N *N*-acyltransferase (AT) from *P. chrysogenum* catalyzes the final step in penicillin biosynthesis. It swaps the hydrophilic side chain of the precursor for various hydrophobic side chains, making it a key enzyme in producing semi-synthetic β-lactam antibiotics. Like other cysteine Ntn-hydrolases, AT is expressed as an inactive precursor that undergoes post-translational self-cleavage for activation, exposing the catalytic N-terminal Cys103 residue. Structural comparison of TANGO2 with AT shows that they share the same overall αββα fold, with an r.m.s.d. of 1.109 Å (between 41 C^α^ atoms), despite having only 12% sequence identity (Fig. 3 ▸*a*). In AT, the residues responsible for catalysis are Cys103, Asn119, Asp121, Asn246 and Arg268 (Bokhove *et al.*, 2010 ▸). These residues are highly conserved in TANGO2 as Cys2, Asn25, Asp27 and Asn157, with the exception that Arg268 is replaced by Lys166. Furthermore, the substrate-binding residues in AT (Trp120, Phe122, Phe123 and Tyr166) are replaced by Arg26, Glu28, Phe29 and Leu73 in TANGO2 (Fig. 3 ▸*b*). The chemically ambiguous nature of the proposed TANGO2 substrate-binding pocket indicates it can accommodate a variety of substrates with different sizes and polarities. However, with only the TANGO2 apo structure available, identifying its native substrates remains challenging due to the complex and versatile nature of the enzyme.

#### 3.4.2. Comparison with *Bifidobacterium longum* bile-salt hydrolase (*Bl*BSH; PDB entry 2hf0)

The bacterial bile-salt hydrolase (BSH) catalyzes the hydrolysis of the amide bond in glycine- or taurine-conjugated bile salts, resulting in the release of free glycine or taurine and bile acids (Funabashi *et al.*, 2020 ▸; Begley *et al.*, 2006 ▸). It was recently demonstrated that BSH not only has hydrolase activity but also exhibits amine *N*-acyltransferase activity (Rimal *et al.*, 2024 ▸). This new finding adds complexity to the functions of cysteine Ntn-hydrolases and broadens our understanding of these enzymes. Although the conjugation mechanism is not yet fully understood, the hydrolytic activities of BSH are well characterized. In *Bl*BSH, the N-terminal cysteine is identified as the key catalytic residue, with several nearby residues also essential for activity, including Arg17, Asp20, Asn81, Asn172 and Arg225 (Kumar *et al.*, 2006 ▸). Structural comparisons between TANGO2 and *Bl*BSH reveal that both proteins share a highly similar αββα core structure, with an r.m.s.d. of 1.267 Å (between 31 C^α^ atoms; Fig. 3 ▸*c*). A significant difference lies in a 32-residue loop (Cys188–Val220) within β-sheet II of *Bl*BSH. The superimposed structures show notable conservation in the positions and orientations of active-site residues. In TANGO2, the N-terminal residue Cys2 corresponds to the catalytic Cys1 in the *Bl*BSH structure. Other catalytically key residues of *Bl*BSH, including Arg17, Asp20, Asn81, Asn172 and Arg225, align well with Arg26, Asp27, Asn75, Asn157 and Lys166 in TANGO2 (Fig. 3 ▸*d*). Compared with the BSH, TANGO2 exhibits significant differences in the putative substrate-binding site, which involves residues in β2 of β-sheet I and β6 of β-sheet II. Moreover, the Trp21 residue in BSH, important for substrate binding and selectivity, is replaced by Phe29 in TANGO2.

#### 3.4.3. Comparison with human acid ceramidase (PDB entry 5u7z)

Human acid ceramidase (AC) hydrolyzes ceramide into fatty acids and sphingosine, which forms the backbone of all sphingolipids and helps to regulate many cellular processes including proliferation, apoptosis, metabolism and inflammation. Although TANGO2 and human AC differ significantly in their amino-acid sequences, structural superposition shows that the αββα fold cores of both proteins align very well, with an r.m.s.d. of 1.179 Å across 56 C^α^ atoms (Fig. 3 ▸*e*). In human AC, residue Asp162 (corresponding to Asp27 in TANGO2) is responsible for stabilizing the positively charged N-terminal amino group of catalytic Cys143 (corresponding to Cys2 in TANGO2) in the hydrolysis reaction, while the oxyanion hole is contributed by the side-chain N atom of Asn320 (corresponding to Asn157 in TANGO2). Arg333 (corresponding to Lys166 in TANGO2) is also important for positioning the catalytic Cys143 and possibly interacting with substrates. These key residues are highly conserved in TANGO2, except for Arg333 in AC, which is replaced by Lys166 in TANGO2. Despite the overall structural similarities, the main differences include the following: (i) a short loop (Pro79–Gln84) between α1 and β4 in TANGO2 is replaced by an α-helix (Gly231–Ile240) in AC, (ii) a bent α-helix (α6, Pro207–Tyr224) present in TANGO2 is missing in AC and (iii) TANGO2 lacks the α-helix (Val177–Leu182) found in AC. These structural elements are in close proximity to the catalytic cysteine and may provide clues about TANGO2 function.

Additionally, the reported hydrophobic substrate-binding pocket of AC, made up of Phe163, Val165, Phe166, Phe227, Phe328, Phe329, Leu367 and Trp395 (Gebai *et al.*, 2018 ▸), is partially replaced by Phe29, Leu77, Ile209, Tyr 224 and Gly235 in TANGO2. Three hydrophobic residues on the AC surface, Phe328, Phe329 and Trp395, overlap with the bent α-helix (α6; Pro207–Tyr224) in TANGO2 (Fig. 3 ▸*f*). This structural difference results in a substantially deeper putative substrate-binding cavity in TANGO2.

#### 3.4.4. Comparison with human *N*-acylethanolamine-hydrolyzing acid amidase (PDB entry 6dxx)

*N*-Acylethanolamines (NAEs) are bioactive lipids that bind to various receptors to exert diverse effects in the nervous and immune systems (Gorelik *et al.*, 2018 ▸). Their activities can be terminated by *N*-acylethanolamine acid amidase (NAAA), which hydrolyzes the amide bond of NAE, releasing free fatty acids (Piomelli *et al.*, 2020 ▸). Although NAAA shares only 12% sequence identity with TANGO2, their αββα fold core structures are highly similar, with an r.m.s.d. of 0.909 Å between 32 C^α^ atoms (Fig. 3 ▸*g*). Structural superposition shows three major differences: (i) TANGO2 has an additional α-helix (α6, Pro207–Tyr224), which is absent in NAAA, (ii) the loop (Pro79–Gln84) between α1 and β4 in TANGO2 is replaced by a hydrophobic α-helix (α6, Trp201–Leu209) in NAAA and (iii) NAAA has only five β-strands in sheet I, two fewer than TANGO2; in particular, the long C-terminal β-strand is missing. Like other cysteine Ntn-hydrolases, the active site of NAAA contains the N-terminal catalytic Cys126 and several important residues, such as Asp145, Asn287 and Arg300, which are highly conserved in TANGO2 (corresponding to Cys2, Asp27, Asn157 and Lys166 in TANGO2). Based on the proposed NAAA mechanism (Gorelik *et al.*, 2018 ▸), substrate binding is triggered by an outward rotation of helix α6, exposing the ‘WWW’ segment (Trp200–Trp202) to form a hydrophobic cavity adjacent to the active site. In TANGO2, the ‘WWW’ segment in NAAA is replaced by a short surface loop (Pro79–Gln84) and Trp201 is substituted by Trp83, which is positioned on the TANGO2 surface (Fig. 3 ▸*h*). This substitution likely increases the conformational flexibility of TANGO2 and the ability to create a more dynamic binding pocket upon substrate binding.

### 3.5. Putative substrates of TANGO2

So far, the function and substrates of TANGO2 remain unknown. Members of the cysteine Ntn-hydrolase family differ considerably in substrate specificity, and most of the substrate-binding mechanisms have not been fully established. Nevertheless, the structural characteristics of TANGO2 strongly indicate the presence of a potential substrate-binding site adjacent to the N-terminal cysteine, an essential feature consistent with other cysteine Ntn-hydrolases.

**Figure 4:**
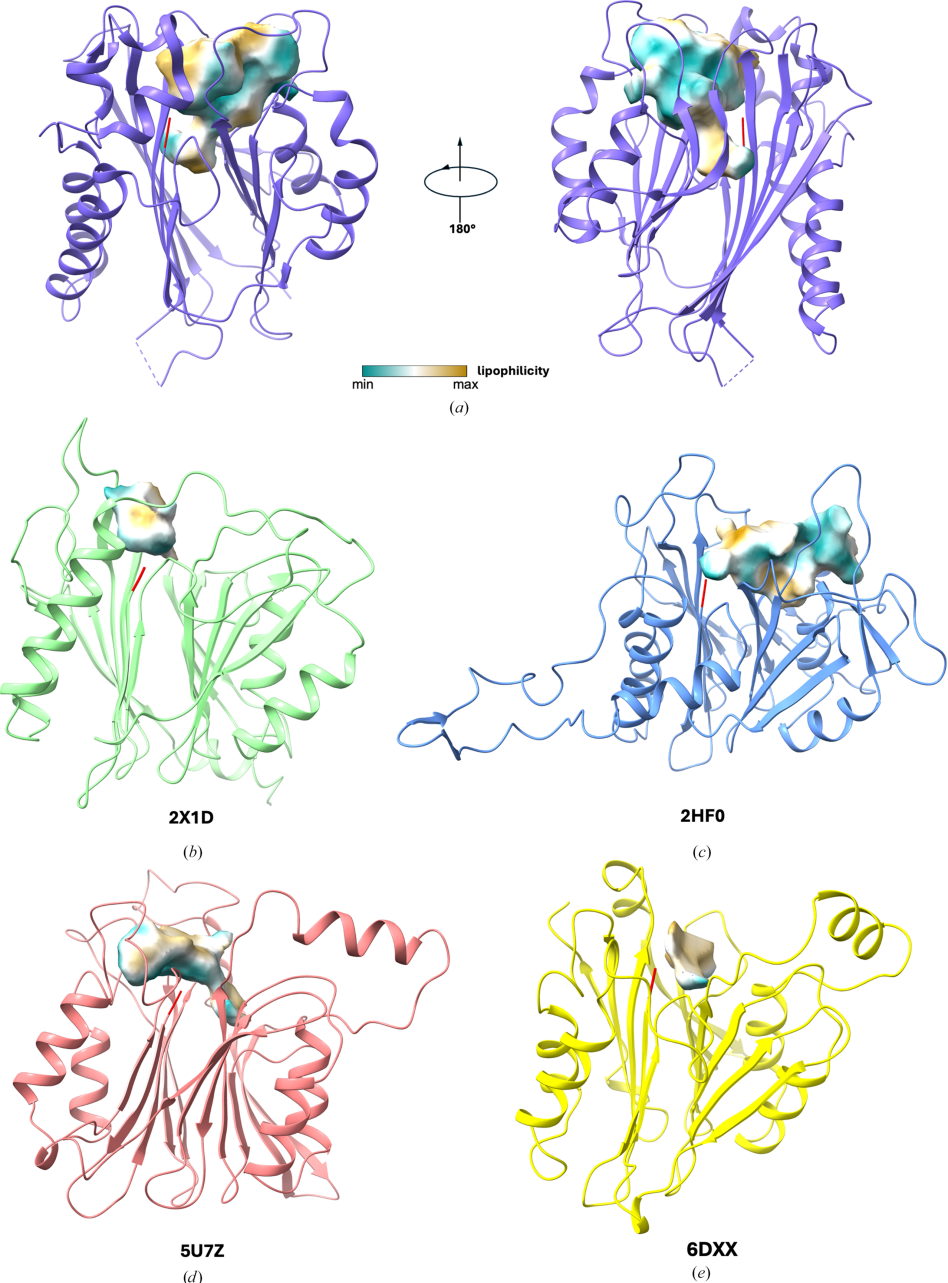
The substrate-binding cavities in TANGO2 and four cysteine Ntn-hydrolases. The overall view of the cavity substructures near the N-terminal cysteines of TANGO2 (*a*) and four cysteine Ntn-hydrolases structures (*b*–*e*). The cavity surface was colored by molecular lipophilic potential (MLP), ranging from dark cyan (most hydrophilic) to white (neutral) to dark goldenrod (most lipophilic). N-terminal cysteines are highlighted in red. The characterizations of each cavity are summarized in Table 2 ▸.

We then analyzed potential substrate-binding cavities in TANGO2 alongside four representative cysteine Ntn-hydrolases and observed that all of the proteins possess cavities of varying volumes adjacent to the N-terminal cysteine residues (Figs. 4 ▸*a*–4*e*). Specifically, TANGO2 possesses the largest putative substrate-binding cavity, with a calculated volume of 956.45 Å^3^. This is followed by bile-salt hydrolase, which has a cavity volume of 899.21 Å^3^, while *N*-acylethanolamine-hydrolyzing acid amidase displays the smallest cavity of 102.82 Å^3^ (see Table 2 ▸ for cavity metrics).

We next investigated whether the volume of the substrate-binding cavity could serve as a predictive parameter for estimating substrate size. This hypothesis, if validated, could provide a structural basis for inferring substrate characteristics in enzymes with unknown specificity. By comparing the molecular weights of known substrates with the corresponding cavity volumes across four cysteine Ntn-hydrolases, we observed a potential positive correlation. The substantial cavity volume observed in TANGO2 suggests its capacity to accommodate substrates exceeding the size of conjugated bile salts, potentially much greater than 500 Da. The conserved cavities observed in TANGO2 and other cysteine Ntn-hydrolases indicate a shared structural feature that facilitates substrate entry and positioning in the active site. This structural feature reinforces the observation that TANGO2 exhibits significant structural homology with other members of the cysteine Ntn-hydrolase family, supporting its classification within this family.

To further characterize the chemical properties of the putative substrate-binding cavity in TANGO2, we performed molecular lipophilic potential (MLP) calculations on the cavity surface. The analysis reveals a heterogeneous distribution of hydrophilic and hydrophobic regions of the cavity, indicative of an amphipathic environment. This dual nature suggests that the cavity could be structurally equipped to accommodate substrates bearing both polar and nonpolar functional groups. Notably, the region surrounding the N-terminal Cys2 exhibits a predominantly hydrophilic character. In contrast, areas close to the protein surface are largely hydrophobic (Fig. 4 ▸*a*). This spatial distribution of polarity may facilitate substrate recognition and orientation. Comparable MLP analyses conducted on other cysteine Ntn-hydrolases reveal polarity distribution patterns that closely resemble those observed in TANGO2. This conservation further supports the structural and functional homology among members of the cysteine Ntn-hydrolase family.

Recently, a lipidomics study on TDD patients showed a marked increase in lysophosphatidic acid (LPA) and a decrease in its precursor phosphatidic acid (PA), indicating that TANGO2 primarily functions in lipid homeostasis (Lujan *et al.*, 2023 ▸; Mehranfar *et al.*, 2024 ▸). Additionally, recent case reports found that vitamin B_5_ can significantly improve the condition of TDD patients (Yılmaz-Gümüş *et al.*, 2023 ▸; Sandkuhler *et al.*, 2023 ▸). In mitochondria, vitamin B_5_ serves as the precursor of coenzyme A (CoA), which is crucial for fatty-acid metabolism and other metabolic pathways. A recent study demonstrated that TANGO2 can bind to CoA and 16- and 18-carbon acyl-CoA species (Lujan *et al.*, 2025 ▸). Taken together, TANGO2 may play a role in regulating the levels of fatty-acid conjugates in lipid metabolism and subcellular lipid-signaling pathways.

**Figure 5:**
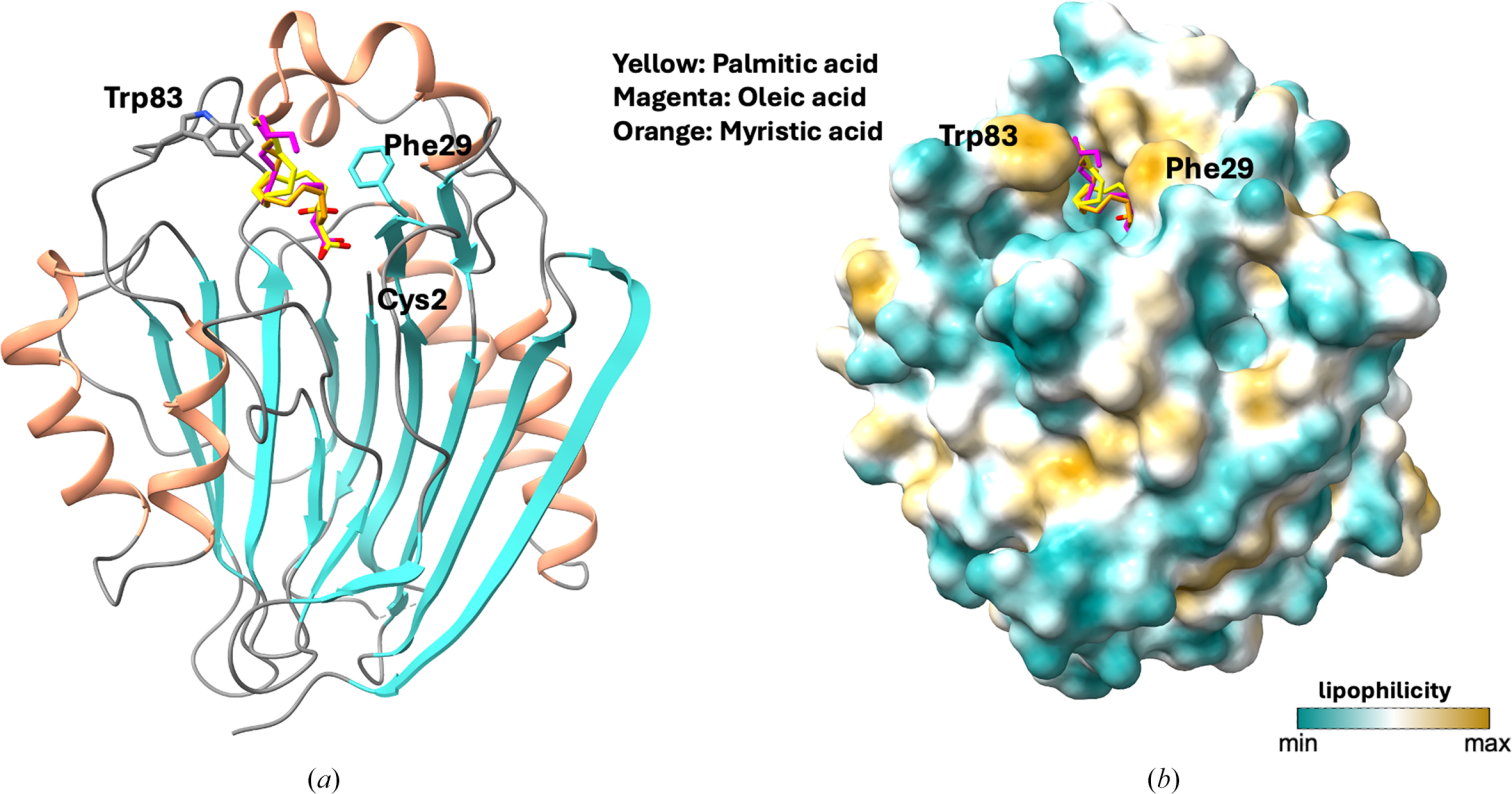
The docked structures of TANGO2 with substrate mimics, including three fatty acids: palmitic acid, oleic acid and myristic acid. (*a*) The putative substrate-binding cavity of TANGO2 with fatty acids bound. (*b*) Molecular lipophilic potential (MLP) surface of TANGO2 with fatty acids bound in the putative substrate-binding cavity; two hydrophobic residues, Phe29 and Trp83, form the entrance on the protein surface.

Because complex structures of TANGO2 with its putative substrates bound are not available, we then carried out molecular docking to dock three different fatty acids, oleic acid, palmitic acid and myristic acid, into the TANGO2 crystal structure (Figs. 5 ▸*a* and 5 ▸*b*). In all docked structures, the three fatty acids are observed in the proposed substrate-binding cavity with a similar orientation and conformation. The carboxyl groups of the fatty acids are deeply buried in the cavity, located within 5.5 Å of the Cys2 residue. The aliphatic chains of the fatty acids extend towards the protein surface. The hydrophobic residues Phe29, Leu81, Trp83, Tyr216, Tyr224 and Tyr234 are located within 5 Å of the docked fatty acids, with Phe29 and Trp83 forming the cavity entrance on the protein surface. These two hydrophobic residues may act as a checkpoint, controlling access based on the size, shape and chemical properties of potential substrates. Interestingly, structural alignment reveals that the surface-exposed Phe29 in TANGO2 is conserved across all four cysteine Ntn-hydrolases (Phe23 in PDB entry 2hf0, Phe123 in PDB entry 2x1d, Phe163 in PDB entry 5u7z and Phe148 in PDB entry 6dxx). This conservation may suggest a potential functional or structural role for Phe29, possibly in substrate recognition or selection at the enzyme surface (Fig. 2 ▸*d*).

### 3.6. Pathogenic TANGO2 variants

If TANGO2 functions as a cysteine Ntn-hydrolase, pathogenic mutations are most likely to impair either the proposed N-terminal Cys2 catalytic site or the substrate-binding region. To test our hypothesis, we generated five *AlphaFold*3 (Abramson *et al.*, 2024 ▸) predicted structural models of TANGO2 pathogenic variants: G154R, T74P, G89C, R26K and F5 (or F6) deletion (Owlett *et al.*, 2024 ▸; de Calbiac *et al.*, 2024 ▸; Schymick *et al.*, 2022 ▸; Baek *et al.*, 2021 ▸; Mingirulli *et al.*, 2020 ▸; Dines *et al.*, 2019 ▸; Lalani *et al.*, 2016 ▸).

**Figure 6:**
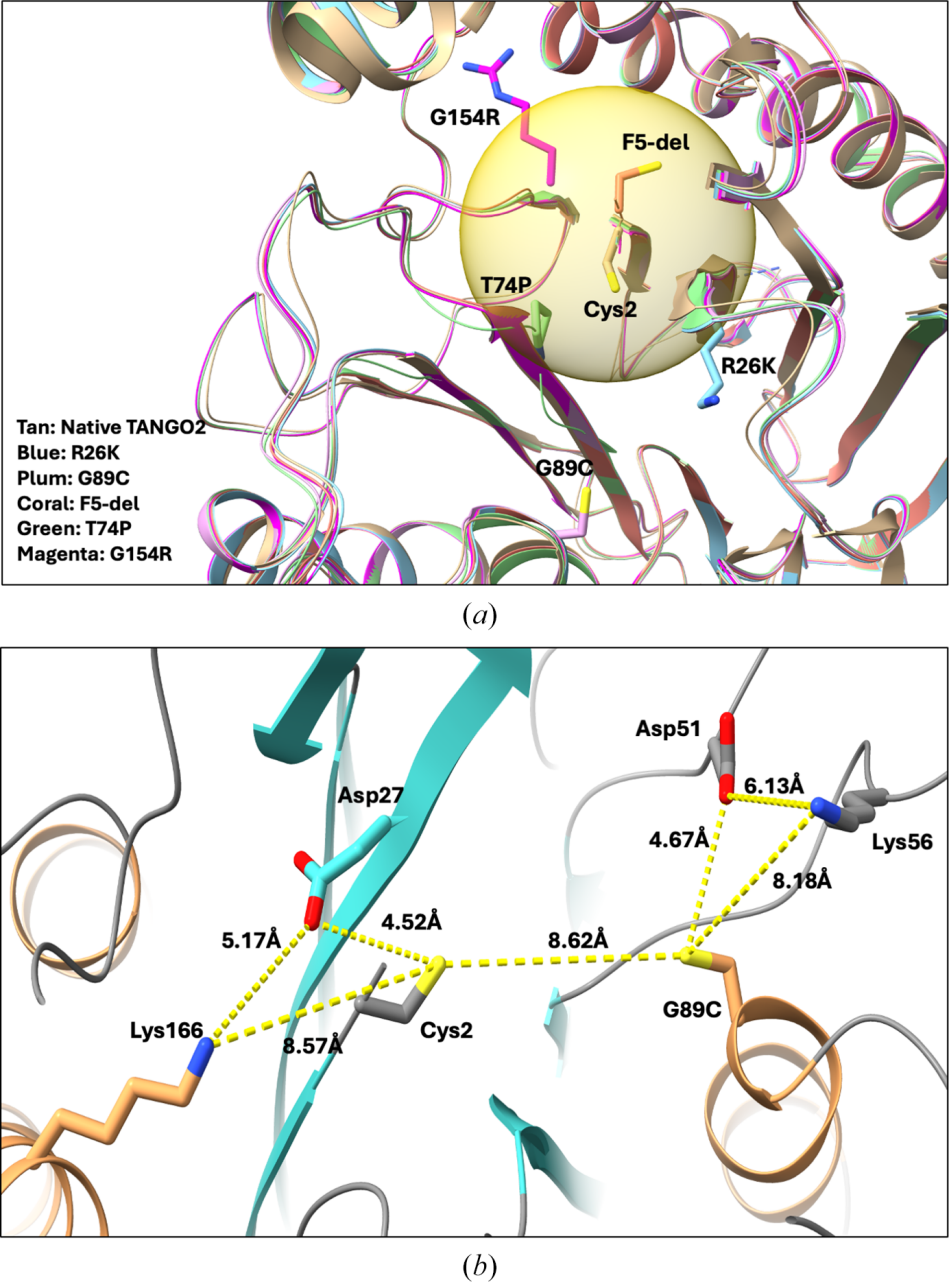
Superposition of the TANGO2 crystal structure with the *AlphaFold*3-predicted structures of pathogenic TANGO2 variants: G154R, T74P, G89C, R26K and F5 deletion. (*a*) The positions of the TANGO2 pathogenic mutations. The yellow circle represents a sphere with a 6.5 Å radius from the N-terminal Cys2. All of the disease-causing mutations are within the sphere, except that G89C is 8.62 Å and G154R is 11.82 Å from the N-terminal Cys2. The side chains of G154R, T74P, G89C and R26K are shown in stick representation; the side chain of the N-terminal cysteine is shown in the structure of the F5 deletion variant. (*b*) In the predicted G89C mutant structure, the side chains of Asp51 and Lys56 are located close to the introduced cysteine residue. This spatial arrangement resembles the configuration of the putative active site, which includes Cys2, Asp27 and Asn157. The G89C mutation may create a pseudo-active site leading to misrecognition or mispositioning of substrates.

Structural comparisons of the five predicted models of TANGO2 mutants with the crystal structure show that (i) all five predicted mutant structures are very similar to the TANGO2 crystal structure, with an r.m.s.d. of less than 0.5 Å (across 271 C^α^ atoms), and (ii) the mutant structures are nearly identical to each other, with an r.m.s.d. under 0.2 Å (across 275 C^α^ atoms). These results indicate that the predicted structures are highly accurate based on the crystal structure, and the mutations in pathogenic variants do not significantly alter the overall structure. However, the mutated residues (T74P, R26K and F5 deletion) are all within a 6.5 Å radius of the proposed catalytic Cys2 (Fig. 6 ▸*a*). Based on our previous analysis, Arg26 is directly involved in the hydrolysis reaction; thus, its mutation could impair TANGO2 enzymatic activity. The G154R and T74P mutations not only change their side-chain sizes but also switch from neutral to hydrophilic. Most cysteine Ntn-hydrolases use hydrophobic substrates and require a hydrophobic binding pocket. These mutations may inhibit the enzyme activity by disrupting the hydrophobic environment of the binding pocket.

Based on the predicted structure, the cysteine side chain of the TANGO2 G89C mutant points towards the Cys2 thiol group, and the distance between the two thiol groups is approximately 8.62 Å. The proximity of two free thiol groups may disrupt the binding and orientation of the substrates. Structural analysis of the G89C mutant reveals that Asp51 and Lys56 are located close to the introduced cysteine residue. This spatial arrangement resembles the configuration of the putative active site, which includes Cys2, Asp27 and Asn157. The similarity in local chemical environment suggests that the G89C substitution may create a pseudo-active site leading to the misrecognition or mispositioning of substrates, potentially interfering with the catalytic function of Cys2. These findings imply that the G89C mutation may compromise the enzymatic activity of TANGO2 by perturbing the structural integrity of the substrate-binding region (Fig. 6 ▸*b*).

The fifth and sixth amino-acid residues in TANGO2 are both phenylalanines; deleting either one results in the same mutant. Based on the predicted structure, removing F5 or F6 moves Cys2 away from the enzymatic active site by one peptide-bond length of 1.32 Å. More importantly, the orientation of the Cys2 side chain will rotate 180°, pointing away from the active site. Therefore, deletion of F5 or F6 could provide direct evidence that Cys2 is essential in TANGO2 activity.

Overall, the pathogenic TANGO2 variants offer valuable insights into its function and help to identify key residues. Using the predicted variant structures and crystal structure, we show that the N-terminal Cys2 and the residues in close proximity could play vital roles in TANGO2 activity.

## 4. Conclusions

In this study, we determined the crystal structure of human TANGO2. The structural homolog search reveals that TANGO2 is very similar to cysteine Ntn-hydrolases, including both the overall fold and active-site residues; they all contain a characteristic αββα fold and an N-terminal cysteine residue. We compare the structure of TANGO2 with four different cysteine Ntn-hydrolases and suggest that TANGO2 functions as a cysteine Ntn-hydrolase using hydrophobic fatty-acid derivatives as substrates based on available structural data. Next, we generated predicted structural models of TANGO2 pathogenic variants and showed that the mutated residues are all located in close proximity to the putative catalytic N-terminal cysteine residue and could interfere with its enzymatic activity or substrate binding. We analyze the putative substrate-binding cavity of TANGO2 and compare it with those of other cysteine Ntn-hydrolases. Our results show that TANGO2 has the largest substrate-binding cavity, and it could accommodate substrates larger than conjugated bile salts. Using molecular docking, we generate structural models of TANGO2 complexed with fatty-acid substrate mimics. All fatty acids, with varying lengths and saturation levels, are found inside the proposed substrate-binding cavity. Overall, based on the structural data and analyses, we suggest that human TANGO2 belongs to the cysteine Ntn-hydrolase family using fatty-acid derivates as substrates and plays a role in lipid metabolism and signaling. Our crystallographic results on the light brown crystals from heme soaking and co-crystallization did not provide direct evidence for a specific TANGO2–heme interaction. However, whether TANGO2 serves dual functions in heme transport and lipid metabolism, and whether these functions are related, remains to be elucidated.

## Supplementary Material

PDB reference: human TANGO2, 8sv7

Supplementary Figures. DOI: 10.1107/S2059798326001968/ag5063sup1.pdf

## Figures and Tables

**Table 1:** Data-collection and refinement statistics for TANGO2 The diffraction data set was collected on beamline 22-ID, SER-CAT, APS (λ = 0.979 Å). Values in parentheses are for the highest resolution shell.

Data collection	
Oscillation range (°)	0.25
Total rotation range (°)	360
Space group	*P*2_1_2_1_2_1_
*a*, *b*, *c* (Å)	49.73, 49.96, 241.38
α, β, γ (°)	90, 90, 90
Resolution (Å)	48.71–1.53 (1.57–1.53)
*R*_merge_	0.046 (0.499)
*R*_p.i.m._	0.018 (0.851)
*R*_meas_	0.048 (0.525)
CC_1/2_	1.00 (0.957)
〈*I*/σ(*I*)〉	28.14 (3.97)
Completeness (%)	94.20 (65.10)
Multiplicity	13.06 (10.01)
Refinement	
No. of reflections	84855 (4390)
*R*_work_/*R*_free_	0.1676/0.1957 (0.2283/0.2291)
No. of atoms	
Total	4771
Protein	4237
Water	524
Ion	10
Wilson *B* factor (Å^2^)	23.72
R.m.s. deviations	
Bond lengths (Å)	0.006
Bond angles (°)	0.85
Ramachandran statistics	
Favored (%)	98.12
Allowed (%)	1.69
Outliers (%)	0.19
Rotamer outliers (%)	0.00
Clashscore	2.75
Average *B* factor (Å^2^)	
Overall	29.98
Macromolecules	29.01
Ligands	38.62
Solvent	37.65
PDB code	8sv7

**Table 2:** Characterization of the cavities near the N-terminal cysteines of TANGO2 and four known cysteine Ntn-hydrolases

Proteins	Volume (Å^3^)	Surface area (Å^2^)	Average depth (Å)	Maximum depth (Å)	Substrates and approximate MW
TANGO2	956.45	698.59	4.44	15.34	?
Bile-salt hydrolase	899.21	605.61	2.96	9.62	Conjugated bile salts, ∼550 Da
Acid ceramidase	362.88	334.81	1.73	6.18	Ceramides, ∼500 Da
Isopenicillin N *N*-acyltransferase	108.36	150.86	1.19	4.02	Isopenicillin N, 359.40 Da
*N*-Acylethanolamine-hydrolyzing acid amidase	102.82	129.21	1.31	3.89	*N*-Acylethanolamines, ∼300 Da

## Data Availability

The atomic coordinates have been deposited in the Protein Data Bank (https://www.pdb.org) as PDB entry 8sv7. Refinement statistics and diffraction data collected on beamline I04 at the Diamond Light Source in the UK are available upon request.

## References

[bb1] Abramson, J., Adler, J., Dunger, J., Evans, R., Green, T., Pritzel, A., Ronneberger, O., Willmore, L., Ballard, A. J., Bambrick, J., Bodenstein, S. W., Evans, D. A., Hung, C.-C., O’Neill, M., Reiman, D., Tunyasuvunakool, K., Wu, Z., Žemgulytė, A., Arvaniti, E., Beattie, C., Bertolli, O., Bridgland, A., Cherepanov, A., Congreve, M., Cowen-Rivers, A. I., Cowie, A., Figurnov, M., Fuchs, F. B., Gladman, H., Jain, R., Khan, Y. A., Low, C. M. R., Perlin, K., Potapenko, A., Savy, P., Singh, S., Stecula, A., Thillaisundaram, A., Tong, C., Yakneen, S., Zhong, E. D., Zielinski, M., Žídek, A., Bapst, V., Kohli, P., Jaderberg, M., Hassabis, D. & Jumper, J. M. (2024). *Nature*, **630**, 493–500.

[bb2] Asadi, P., Milev, M. P., Saint-Dic, D., Gamberi, C. & Sacher, M. (2023). *J. Inher Metab. Dis.* **46**, 358–368. 10.1002/jimd.12579 PMC10464931 36502486

[bb3] Baek, M., DiMaio, F., Anishchenko, I., Dauparas, J., Ovchinnikov, S., Lee, G. R., Wang, J., Cong, Q., Kinch, L. N., Schaeffer, R. D., Millán, C., Park, H., Adams, C., Glassman, C. R., DeGiovanni, A., Pereira, J. H., Rodrigues, A. V., van Dijk, A. A., Ebrecht, A. C., Opperman, D. J., Sagmeister, T., Buhlheller, C., Pavkov-Keller, T., Rathina­swamy, M. K., Dalwadi, U., Yip, C. K., Burke, J. E., Garcia, K. C., Grishin, N. V., Adams, P. D., Read, R. J. & Baker, D. (2021). *Science*, **373**, 871–876. 10.1126/science.abj8754 PMC7612213 34282049

[bb4] Bard, F., Casano, L., Mallabiabarrena, A., Wallace, E., Saito, K., Kitayama, H., Guizzunti, G., Hu, Y., Wendler, F., DasGupta, R., Perrimon, N. & Malhotra, V. (2006). *Nature*, **439**, 604–607. 10.1038/nature04377 16452979

[bb5] Begley, M., Hill, C. & Gahan, C. G. (2006). *Appl. Environ. Microbiol.* **72**, 1729–1738. 10.1128/AEM.72.3.1729-1738.2006 PMC1393245 16517616

[bb6] Bokhove, M., Yoshida, H., Hensgens, C. M. H., Metske van der Laan, J., Sutherland, J. D. & Dijkstra, B. W. (2010). *Structure*, **18**, 301–308. 10.1016/j.str.2010.01.005 20223213

[bb7] Brannigan, J. A., Dodson, G., Duggleby, H. J., Moody, P. C. E., Smith, J. L., Tomchick, D. R. & Murzin, A. G. (1995). *Nature,* **378**, 416–419. 10.1038/378416a0 7477383

[bb8] Carfi, A., Pares, S., Duée, E., Galleni, M., Duez, C., Frère, J.-M. & Dideberg, O. (1995). *EMBO J.* **14**, 4914–4921. 10.1002/j.1460-2075.1995.tb00174.x PMC394593 7588620

[bb9] Cooper, A., Powers, A., Battaile, K. P., Mohsen, A., Johnson, D. K., Lovell, S. & Ghaloul-Gonzalez, L. (2025). *Proteins*, **94**, 515–528. 10.1002/prot.70023 PMC12359130 40726205

[bb10] de Calbiac, H., Montealegre, S., Straube, M., Renault, S., Debruge, H., Chentout, L., Ciura, S., Imbard, A., Le Guillou, E., Marian, A., Goudin, N., Caccavelli, L., Fabrega, S., Hubas, A., van Endert, P., Dupont, N., Diana, J., Kabashi, E. & de Lonlay, P. (2024). *Autophagy Rep.* **3**, 2306766. 10.1080/27694127.2024.2306766 PMC7617261 39722856

[bb11] Dines, J. N., Golden-Grant, K., LaCroix, A., Muir, A. M., Cintrón, D. L., McWalter, K., Cho, M. T., Sun, A., Merritt, J. L., Thies, J., Niyazov, D., Burton, B., Kim, K., Fleming, L., Westman, R., Karachunski, P., Dalton, J., Basinger, A., Ficicioglu, C., Helbig, I., Pendziwiat, M., Muhle, H., Helbig, K. L., Caliebe, A., Santer, R., Becker, K., Suchy, S., Douglas, G., Millan, F., Begtrup, A., Monaghan, K. G. & Mefford, H. C. (2019). *Genet. Med.* **21**, 601–607.

[bb12] Eberhardt, J., Santos-Martins, D., Tillack, A. F. & Forli, S. (2021). *J. Chem. Inf. Model.* **61**, 3891–3898. 10.1021/acs.jcim.1c00203 PMC10683950 34278794

[bb13] Emsley, P., Lohkamp, B., Scott, W. G. & Cowtan, K. (2010). *Acta Cryst.* D**66**, 486–501. 10.1107/S0907444910007493 PMC2852313 20383002

[bb14] Foley, M. H., Walker, M. E., Stewart, A. K., O’Flaherty, S., Gentry, E. C., Patel, S., Beaty, V. V., Allen, G., Pan, M., Simpson, J. B., Perkins, C., Vanhoy, M. E., Dougherty, M. K., McGill, S. K., Gulati, A. S., Dorrestein, P. C., Baker, E. S., Redinbo, M. R., Barrangou, R. & Theriot, C. M. (2023). *Nat. Microbiol.* **8**, 611–628. 10.1038/s41564-023-01337-7 PMC10066039 36914755

[bb15] Funabashi, M., Grove, T. L., Wang, M., Varma, Y., McFadden, M. E., Brown, L. C., Guo, C., Higginbottom, S., Almo, S. C. & Fischbach, M. A. (2020). *Nature*, **582**, 566–570. 10.1038/s41586-020-2396-4 PMC7319900 32555455

[bb16] Gebai, A., Gorelik, A., Li, Z., Illes, K. & Nagar, B. (2018). *Nat. Commun.* **9**, 1621. 10.1038/s41467-018-03844-2 PMC5915598 29692406

[bb17] Gorelik, A., Gebai, A., Illes, K., Piomelli, D. & Nagar, B. (2018). *Proc. Natl Acad. Sci. USA*, **115**, E10032–E10040. 10.1073/pnas.1811759115 PMC6205464 30301806

[bb18] Guerra, J., Ribeiro-Filho, H. V., Jara, G. E., Bortot, L. O., Pereira, J. G. C. & Lopes-de-Oliveira, P. S. (2021). *BMC Bioinformatics*, **22**, 607. 10.1186/s12859-021-04519-4 PMC8685811 34930115

[bb19] Han, S., Guo, K., Wang, W., Tao, Y. J. & Gao, H. (2023). *mBio*, **14**, e0132023. 10.1128/mbio.01320-23 PMC10470608 37462360

[bb20] Heiman, P., Mohsen, A. W., Karunanidhi, A., St Croix, C., Watkins, S., Koppes, E., Haas, R., Vockley, J. & Ghaloul-Gonzalez, L. (2022). *Sci. Rep.* **12**, 3045. 10.1038/s41598-022-07076-9 PMC8866466 35197517

[bb21] Holm, L., Laiho, A., Törönen, P. & Salgado, M. (2023). *Protein Sci.* **32**, e4519. 10.1002/pro.4519 PMC9793968 36419248

[bb22] Jayaram, D. T., Sivaram, P., Biswas, P., Dai, Y., Sweeny, E. A. & Stuehr, D. J. (2025). *Nat. Commun.* **16**, 7972. 10.1038/s41467-025-62819-2 PMC12381273 40858607

[bb23] Jennions, E., Hedberg-Oldfors, C., Berglund, A. K., Kollberg, G., Törnhage, C. J., Eklund, E. A., Oldfors, A., Verloo, P., Vanlander, A. V., De Meirleir, L., Seneca, S., Sterky, F. H. & Darin, N. (2019). *J. Inherit. Metab. Dis.* **42**, 898–908. 10.1002/jimd.12149 31276219

[bb24] Jumper, J., Evans, R., Pritzel, A., Green, T., Figurnov, M., Ronneberger, O., Tunyasuvunakool, K., Bates, R., Žídek, A., Potapenko, A., Bridgland, A., Meyer, C., Kohl, S. A. A., Ballard, A. J., Cowie, A., Romera-Paredes, B., Nikolov, S., Jain, R., Adler, J., Back, T., Petersen, S., Reiman, D., Clancy, E., Zielinski, M., Steinegger, M., Pacholska, M., Berghammer, T., Bodenstein, S., Silver, D., Vinyals, O., Senior, A. W., Kavukcuoglu, K., Kohli, P. & Hassabis, D. (2021). *Nature*, **596**, 583–589. 10.1038/s41586-021-03819-2 PMC8371605 34265844

[bb25] Karlov, D. S., Long, S. L., Zeng, X., Xu, F., Lal, K., Cao, L., Hayoun, K., Lin, J., Joyce, S. A. & Tikhonova, I. G. (2023). *Structure*, **31**, 629–638. 10.1016/j.str.2023.02.014 36963397

[bb26] Kim, E. S., Casey, J. G., Tao, B. S., Mansur, A., Mathiyalagan, N., Wallace, E. D., Ehrmann, B. M. & Gupta, V. A. (2023). *Dis. Models Mech.* **16**, dmm050092. 10.1242/dmm.050092 PMC10499024 37577943

[bb27] Kremer, L. S., Distelmaier, F., Alhaddad, B., Hempel, M., Iuso, A., Küpper, C., Mühlhausen, C., Kovacs-Nagy, R., Satanovskij, R., Graf, E., Berutti, R., Eckstein, G., Durbin, R., Sauer, S., Hoffmann, G. F., Strom, T. M., Santer, R., Meitinger, T., Klopstock, T., Prokisch, H. & Haack, T. B. (2016). *Am. J. Hum. Genet.* **98**, 358–362. 10.1016/j.ajhg.2015.12.009 PMC4746337 26805782

[bb28] Kumar, R. S., Brannigan, J. A., Prabhune, A. A., Pundle, A. V., Dodson, G. G., Dodson, E. J. & Suresh, C. G. (2006). *J. Biol. Chem.* **281**, 32516–32525. 10.1074/jbc.M604172200 16905539

[bb29] Lalani, S. R., Liu, P., Rosenfeld, J. A., Watkin, L. B., Chiang, T., Leduc, M. S., Zhu, W., Ding, Y., Pan, S., Vetrini, F., Miyake, C. Y., Shinawi, M., Gambin, T., Eldomery, M. K., Akdemir, Z. H., Emrick, L., Wilnai, Y., Schelley, S., Koenig, M. K., Memon, N., Farach, L. S., Coe, B. P., Azamian, M., Hernandez, P., Zapata, G., Jhangiani, S. N., Muzny, D. M., Lotze, T., Clark, G., Wilfong, A., Northrup, H., Adesina, A., Bacino, C. A., Scaglia, F., Bonnen, P. E., Crosson, J., Duis, J., Maegawa, G. H., Coman, D., Inwood, A., McGill, J., Boerwinkle, E., Graham, B., Beaudet, A., Eng, C. M., Hanchard, N. A., Xia, F., Orange, J. S., Gibbs, R. A., Lupski, J. R. & Yang, Y. (2016). *Am. J. Hum. Genet.* **98**, 347–357. 10.1016/j.ajhg.2015.12.008 PMC4746334 26805781

[bb30] Liebschner, D., Afonine, P. V., Baker, M. L., Bunkóczi, G., Chen, V. B., Croll, T. I., Hintze, B., Hung, L.-W., Jain, S., McCoy, A. J., Moriarty, N. W., Oeffner, R. D., Poon, B. K., Prisant, M. G., Read, R. J., Richardson, J. S., Richardson, D. C., Sammito, M. D., Sobolev, O. V., Stockwell, D. H., Terwilliger, T. C., Urzhumtsev, A. G., Videau, L. L., Williams, C. J. & Adams, P. D. (2019). *Acta Cryst.* D**75**, 861–877. 10.1107/S2059798319011471 PMC6778852 31588918

[bb31] Linhorst, A. & Lübke, T. (2022). *Cells*, **11**, 1592. 10.3390/cells11101592 PMC9140057 35626629

[bb32] Lujan, A. L., Foresti, O., Sugden, C., Brouwers, N., Farre, A. M., Vignoli, A., Azamian, M., Turner, A., Wojnacki, J. & Malhotra, V. (2023). *eLife*, **12**, e85345. 10.7554/eLife.85345 PMC10042531 36961129

[bb33] Lujan, A. L., Foresti, O., Wojnacki, J., Bigliani, G., Brouwers, N., Pena, M. J., Androulaki, S., Hashidate-Yoshida, T., Kalyukina, M., Novoselov, S. S., Shindou, H. & Malhotra, V. (2025). *J. Cell Biol.* **224**, e202410001. 10.1083/jcb.202410001 PMC11867700 40015245

[bb34] Mehranfar, M., Asadi, P., Shokohi, R., Milev, M. P., Gamberi, C. & Sacher, M. (2024). *Biochem. Biophys. Res. Commun.* **717**, 150047. 10.1016/j.bbrc.2024.150047 38718569

[bb35] Milev, M. P., Saint-Dic, D., Zardoui, K., Klopstock, T., Law, C., Distelmaier, F. & Sacher, M. (2021). *J. Inher Metab. Dis.* **44**, 426–437. 10.1002/jimd.12312 32909282

[bb36] Mingirulli, N., Pyle, A., Hathazi, D., Alston, C. L., Kohlschmidt, N., O’Grady, G., Waddell, L., Evesson, F., Cooper, S. B. T., Turner, C., Duff, J., Topf, A., Yubero, D., Jou, C., Nascimento, A., Ortez, C., García-Cazorla, A., Gross, C., O’Callaghan, M., Santra, S., Preece, M. A., Champion, M., Korenev, S., Chronopoulou, E., Anirban, M., Pierre, G., McArthur, D., Thompson, K., Navas, P., Ribes, A., Tort, F., Schlüter, A., Pujol, A., Montero, R., Sarquella, G., Lochmüller, H., Jiménez-Mallebrera, C., Taylor, R. W., Artuch, R., Kirschner, J., Grünert, S. C., Roos, A. & Horvath, R. (2020). *J. Inher Metab. Dis.* **43**, 297–308. 10.1002/jimd.12156 PMC7078914 31339582

[bb37] Minor, W., Cymborowski, M., Otwinowski, Z. & Chruszcz, M. (2006). *Acta Cryst.* D**62**, 859–866. 10.1107/S0907444906019949 16855301

[bb38] Miyake, C. Y., Lay, E. J., Soler-Alfonso, C., Glinton, K. E., Houck, K. M., Tosur, M., Moran, N. E., Stephens, S. B., Scaglia, F., Howard, T. S., Kim, J. J., Pham, T. D., Valdes, S. O., Li, N., Murali, C. N., Zhang, L., Kava, M., Yim, D., Beach, C., Webster, G., Liberman, L., Janson, C. M., Kannankeril, P. J., Baxter, S., Singer-Berk, M., Wood, J., Mackenzie, S. J., Sacher, M., Ghaloul-Gonzalez, L., Pedroza, C., Morris, S. A., Ehsan, S. A., Azamian, M. S. & Lalani, S. R. (2023). *Genet. Med.* **25**, 100352. 10.1016/j.gim.2022.11.020 PMC10306319 36473599

[bb39] Oinonen, C. & Rouvinen, J. (2000). *Protein Sci.* **9**, 2329–2337. 10.1110/ps.9.12.2329 PMC2144523 11206054

[bb40] Owlett, L. D., Zapanta, B., Sandkuhler, S. E., Ames, E. G., Hickey, S. E., Mackenzie, S. J. & Meisner, J. K. (2024). *Am. J. Med. Genet. A*, **194**, e63778. 10.1002/ajmg.a.63778 PMC11502271 38829177

[bb41] Pettersen, E. F., Goddard, T. D., Huang, C. C., Couch, G. S., Greenblatt, D. M., Meng, E. C. & Ferrin, T. E. (2004). *J. Comput. Chem.* **25**, 1605–1612. 10.1002/jcc.20084 15264254

[bb42] Pettersen, E. F., Goddard, T. D., Huang, C. C., Meng, E. C., Couch, G. S., Croll, T. I., Morris, J. H. & Ferrin, T. E. (2021). *Protein Sci.* **30**, 70–82. 10.1002/pro.3943 PMC7737788 32881101

[bb43] Piomelli, D., Scalvini, L., Fotio, Y., Lodola, A., Spadoni, G., Tarzia, G. & Mor, M. (2020). *J. Med. Chem.* **63**, 7475–7490. 10.1021/acs.jmedchem.0c00191 32191459

[bb44] Powers, A. E. (2024). Masters thesis, University of Pittsburgh, USA.

[bb45] Rimal, B., Collins, S. L., Tanes, C. E., Rocha, E. R., Granda, M. A., Solanki, S., Hoque, N. J., Gentry, E. C., Koo, I., Reilly, E. R., Hao, F., Paudel, D., Singh, V., Yan, T., Kim, M. S., Bittinger, K., Zackular, J. P., Krausz, K. W., Desai, D., Amin, S., Coleman, J. P., Shah, Y. M., Bisanz, J. E., Gonzalez, F. J., Vanden Heuvel, J. P., Wu, G. D., Zemel, B. S., Dorrestein, P. C., Weinert, E. E. & Patterson, A. D. (2024). *Nature*, **626**, 859–863. 10.1038/s41586-023-06990-w PMC10881385 38326609

[bb46] Sacher, M., DeLoriea, J., Mehranfar, M., Casey, C., Naaz, A., Mackenzie, S. J. & Gamberi, C. (2024). *Dis. Models Mech.* **17**, dmm050662. 10.1242/dmm.050662 PMC11179719 38836374

[bb47] Sandkuhler, S. E. & Mackenzie, S. J. (2025). *J. Cell Biol.* **224**, e202503010. 10.1083/jcb.202503010 PMC11998701 40232182

[bb48] Sandkuhler, S. E., Youngs, K. S., Gottipalli, O., Owlett, L. D., Bandora, M. B., Naaz, A., Kim, E., Wang, L., Wojtovich, A., Gupta, V., Sacher, M. & Mackenzie, S. J. (2025). *eLife*, **14**, RP105418. 10.7554/eLife.105418 PMC12782551 41504601

[bb49] Sandkuhler, S. E., Zhang, L., Meisner, J. K., Ghaloul–Gonzalez, L., Beach, C. M., Harris, D., de Lonlay, P., Lalani, S. R., Miyake, C. Y. & Mackenzie, S. J. (2023). *J. Inher Metab. Dis.* **46**, 161–162. 10.1002/jimd.12585 PMC10204720 36550018

[bb50] Schymick, J., Leahy, P., Cowan, T., Ruzhnikov, M. R. Z., Gates, R., Fernandez, L., Pramanik, G., Undiagnosed Diseases Network, Yarlagadda, V., Wheeler, M., Bernstein, J. A., Enns, G. M. & Lee, C. (2022). *Am. J. Med. Genet. A*, **188**, 473–487. 10.1002/ajmg.a.62543 PMC13739840 34668327

[bb51] Stentenbach, M., Hughes, L. A., Fagan, S. V., Payne, B., Rudler, D. L., Siira, S. J., McCubbin, T., Chopin, A., Perks, K. L., Ermer, J. A., Hendry, J., Er, T. S., Balasubramaniam, S., Eliades, J. A., Hool, L. C., Packer, N. H., Moh, E. S. X., Padman, B. S., Rackham, O. & Filipovska, A. (2025). *Nat. Commun.* **16**, 5261. 10.1038/s41467-025-60563-1 PMC12144310 40480980

[bb52] Sun, F., Zhao, Z., Willoughby, M. M., Shen, S., Zhou, Y., Shao, Y., Kang, J., Chen, Y., Chen, M., Yuan, X., Hamza, I., Reddi, A. R. & Chen, C. (2022). *Nature*, **610**, 768–774. 10.1038/s41586-022-05347-z PMC9810272 36261532

[bb53] Suresh, C. G., Brannigan, J. A., Pundle, A. V., SivaRaman, H., Rao, K. N., McVey, C. E., Verma, C. S., Dauter, Z., Dodson, E. J. & Dodson, G. G. (1999). *Nat. Struct. Biol.* **6**, 414–416. 10.1038/8213 10331865

[bb54] van Kempen, M., Kim, S. S., Tumescheit, C., Mirdita, M., Lee, J., Gilchrist, C. L. M., Söding, J. & Steinegger, M. (2024). *Nat. Biotechnol.* **42**, 243–246. 10.1038/s41587-023-01773-0 PMC10869269 37156916

[bb55] Yılmaz-Gümüş, E., Elcioglu, N. H., Genç, E., Arıcı, Ş., Öztürk, G., Yapıcı, Ö., Akalın, F. & Öztürk-Hişmi, B. (2023). *J. Pediatr. Endocrinol. Metab.* **36**, 983–987. 10.1515/jpem-2023-0172 37381587

