# Supplementary material for: The crystal structure of human transport and Golgi organization 2 homolog (TANGO2) suggests a cysteine N-terminal nucleophile (Ntn) hydrolase
Source: Acta Crystallogr D Struct Biol. 2026 Apr 1;82(Pt 4):383–96. doi: 10.1107/S2059798326001968 (PMC13044924; doi:10.1107/S2059798326001968)
Supplement: Supplementary file 1 [file d-82-00383-sup1.pdf]

Acta Cryst D

STRUCTURAL BIOLOGY

Volume 82 (2026)

Supporting information for article:

The crystal structure of human transport and Golgi organization 2 homolog (TANGO2) suggests a cysteine N-terminal nucleophile (Ntn) hydrolase

Dayong Zhou, Lirong Chen, John Rose and Bi-Cheng Wang

```
TANGO2/1-276  M..CIIFFKFD.PRPVSKNAY..R.LILAANRDEF..............Y..SR..PSKL
2HF0/1-316    ...CTGVR.F.........SDDEGNTYFGRNLDWS..............FS..Y..GE.T
2X1D/103-356  ...CTTAYCQL...P....NG....ALQGQNWDFF..............S..ATKENLI.
6DXX/126-359  ...CTSIVAQDS..R....G...H.IYHGRNLDYP..............FGNVLRKLT.V
5U7Z/141-395  .TICTSIVAEDK..K....GH....LIHGRNMDFGVFLGWNINNDTWVIT.EQLKPLT.V

TANGO2/1-276  ADFWGNN..................NEILSGLD..MEEGKE...G.GTWLGISTRGKLAA
2HF0/1-316    ILVTPRGYHYDTVFGAGGKAKPN...AVIGVGV.V.....MADRP.MYFDCANEHGLAIA
2X1D/103-356  RLTIRQA.............GLP...TIKFITE..A....G...IIGK.VGFNSAGVAVN
6DXX/126-359  DVQFLKN................GQIAFTGTTFIGY........V.GLWTGQSPHKFTVS
5U7Z/141-395  NLDFQRN................NKTVFKASSFAGY........V.GMLTGFKPGLFSLT

TANGO2/1-276  LTNYLQ......PQL.D...WQA.................RGRGELVTHFLTTDV....D
2HF0/1-316    GLNF.PGY..AS.......F...V..H....EPVEGTENVA.TFEFPLWVARN.FDSVDE
2X1D/103-356  YNAL.H.L.Q.G.......L...R..P....T......GVP.SHIALRIALE..STSPSQ
6DXX/126-359  GDER.DK..G....W.WWEN.A..I.AALF.RR.....H.IPVSWLIRATLSE.SENFEA
5U7Z/141-395  LNER.FS.IN.G...GY.LG..I.LEW.ILGKKD....AMW.IGFLTRTVLEN.STSYEE

TANGO2/1-276  S.L.SYL.K.KVSMEGHLY.........NGFNLIAADLSTAKGD..VICYYGNR......
2HF0/1-316    V.EET...LRNV..TL.VSQIVPGQQESL.LHWFIGDG.....K..RSIVVEQM.ADGMH
2X1D/103-356  AY..DRI.V.EQ.GGM.AA..........SAFIMVGNG.....H..EAFGLEFSP..TSI
6DXX/126-359  AVG..KLA...K..TP.LI.........ADVYYIVGGT.....SPREGVVITRNR..DGP
5U7Z/141-395  AKN..LLTK..T..KI.LA..........PAYFILGGN.....QSGEGCVITRDR..KES

TANGO2/1-276  GEPDP.I.VL.T.P.....GTYGLSNA.LLET....P.......................
2HF0/1-316    ....V.H..HDD........VDVLTNQPT..FDF..H.................MENLRN
2X1D/103-356  .R....KQ.V.L.D...ANGRMVHTNH.C..LL.....QHGKNEKELDPLP.........
6DXX/126-359  AD....IW.P.LDP.LNGAWFRVETNY.D..H.WKPAP..............KE......
5U7Z/141-395  .L...DVY.E.L.DAKQGRWYVVQTNY.D..R.WK.HP............FF.L......

TANGO2/1-276  .......................................WRKLCFGKQLFL..E...AV.
2HF0/1-316    YMCVSNEMAEPTSWGKASLTAWGAGVGMHGIPGDV..SSPSRFVRVAY..T...NAH.Y.
2X1D/103-356  ..................................DSWN...RHQRMEF.LLDGF......
6DXX/126-359  ..................................D..D...RRTSAIK.ALNAT....GQ
5U7Z/141-395  ..................................D..D...RRTPAKMCL.NRT..S.Q.

TANGO2/1-276  ..E.RSQALP....K..DVLIAS..L..LDVLNNEE......AQLPDPAIEDQGGEYVQP
2HF0/1-316    .P.Q......QNDEAAN..VS.RLFHTL..G..S..VQMVDGM.................
2X1D/103-356  ..DG.T.............KQ.A.FA...QLWADED......N.................
6DXX/126-359  AN.L............S..LE.ALFQIL..S..VVPVYN.....................
5U7Z/141-395  .ENI............S..FE.T.M...YDVLSTK.........................

TANGO2/1-276  MLS..KYAAVCVR..........CPGYGTRTNTIILVDADG..H.V.TFTERSM...MDK
2HF0/1-316    .....A...........KMGDGQF..ERTLFTSGYSS......K.TNTYYMNT.Y.....
2X1D/103-356  ...YPF..SICRAYEEGK.....SR.GATLFNIIYDHA......RR.EATVRLGRPT...
6DXX/126-359  ............N..............FTIYTTVM.S....AGS.PDKYMTRI.R.....
5U7Z/141-395  ........PVLNK..............LTVYTTLIDV......TKG.QFETYL.R.....

TANGO2/1-276  DLSHWE..TRTYEF.........TLQS.........................
2HF0/1-316    .D.D..PAIRSYAMADY.DM..D....SSELISVAR................
2X1D/103-356  .N.PDE..MFVMRFD..EEDERS.............ALNAR...........
6DXX/126-359  .N.P.....................................SRK........
5U7Z/141-395  .D..........................................CPDPCIGW
```

Figure S1 Amino acid sequence alignment between TANGO2 (PDB 8SV7) and four cysteine Ntn-hydrolases (PDB 2HF0, 2X1D, 6DXX, and 5U7Z). Shown on the left are the PDB entries and residue numbers of each protein. The identical amino acid residues among all the members are highlighted in red, those with strong conservation are highlighted in cyan.

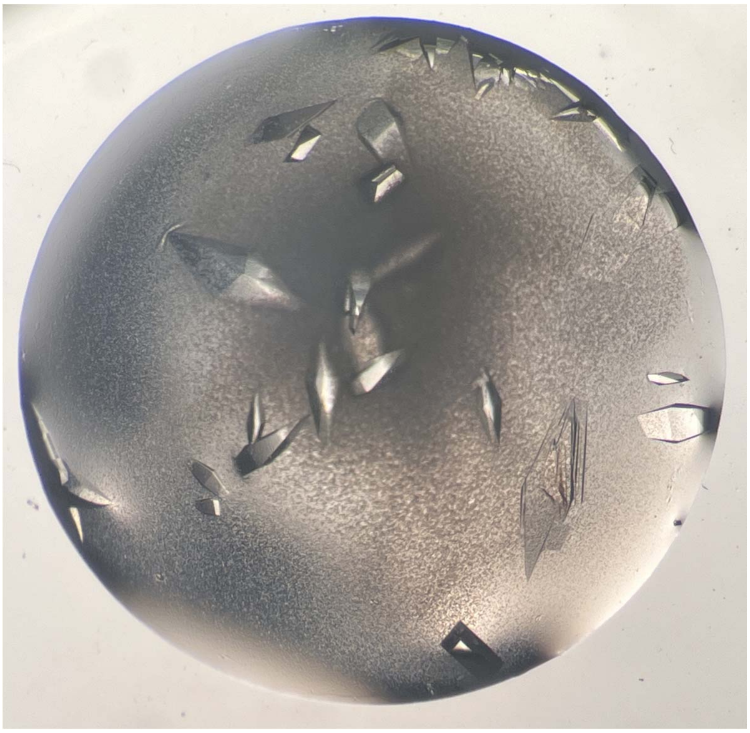

Figure S2 Crystals of TANGO2 grew in 0.1M Bis-Tris pH 5.5, 1.0 M Ammonium sulfate, and 1% PEG 3350.
